# A systematic review of electron FLASH dosimetry and beam control mechanisms utilized with modified non‐clinical LINACs

**DOI:** 10.1002/acm2.70051

**Published:** 2025-03-19

**Authors:** Justin DeFrancisco, Siyong Kim

**Affiliations:** ^1^ Medical Physics Program Virginia Commonwealth University Richmond Virginia USA; ^2^ Department of Radiation Oncology School of Medicine Virginia Commonwealth University Richmond Virginia USA

**Keywords:** detector, dosimetry, electron, FLASH, LINAC, UHDR

## Abstract

**Background:**

FLASH has been shown to spare normal tissue toxicity while maintaining tumor control. However, existing irradiation platforms and dosimetry are not compatible. Consequently, an abundance of FLASH delivery devices and new dosimetry across all modalities has been created. Many review articles concluded that dosimetry is modality‐dependent. Focusing on electrons, researchers have modified clinical LINACs to enable FLASH dose rates. Modified LINACs caused the development of unique control systems that have yet to be characterized. Improvement could be made when considering the organization of reviews.

**Purpose:**

To systematically perform a literature survey on electron FLASH dosimetry and beam control mechanisms with modified LINACs, detail where articles originated, and organize the results.

**Methods:**

A literature survey was performed from two websites using specified keywords and sifted results to find articles that fit the criteria. The results were organized in tables and summaries effectively by matching up dosimeters with their measurement goal, referring to their specific models, outlining the irradiation conditions they were tested in, and detailing their calibration procedure. Furthermore, included was the unique topic of control mechanisms.

**Results:**

Twenty‐eight matches were found. Various dosimeters were examined to measure absorbed dose, beam characteristics (BC), dose per pulse (DPP), and pulse counting (PC). Specific detectors and the irradiation conditions are organized and presented in a table. Each model's pros and cons are presented in another table for further consideration. A third table is provided to detail beam control methods.

**Conclusions:**

Dosimetry is majorly film‐based for absorbed dose and beam characteristic measurements. Many candidates for dosimeters for the use of DPP and PC have been tested, but they have yet to be tested without limitations. Beam control mechanisms primarily consist of unacceptable delivery errors. Many suggestions for improvement were given, mainly consisting of finding new dosimeters and modulating the dose DPP.

## 1 INTRODUCTION

Conventionally, external beam radiotherapy utilizes low‐dose‐rate irradiation techniques, achieving a maximum of 6 Gy/min.^1^ These dose rates have been widely accepted as the optimal method until the current decade. Favaudon et al.^2^ rediscovered that raising the dose rate to > 40 Gy/s (FLASH or ultra‐high dose rate, UHDR) decreased the normal tissue complication probability without affecting the tumor control. There is an abundance of research confirming the FLASH effect biologically.^2^, ^3^, ^4^, ^5^ Due to the great number of findings, several notable FLASH effect reviews have already been published.^6^, ^7^, ^8^, ^9^, ^10^ Confirmation of this effect is still widely ongoing due to challenging obstacles that need to be overcome to make FLASH applicable. Current dosimetry techniques and delivery devices are no longer compatible with UHDRs. In an attempt to harness this normal tissue‐sparing effect, many researchers have begun studying solutions. FLASH has been achieved for several radiation modalities, such as protons, heavy ions, photons, and electrons. For dosimetry solutions, hundreds of studies already warranted the formation of several review articles. The most notable were conducted by Ashraf et al.,^11^ Romano et al.,^12^ and Siddique et al.^13^ These reviews present compatible detector classes, explain the operational mechanisms of each class, and outline the determined properties in UHDRs. However, these reports are comprehensive, covering all dosimetry methods across every modality of FLASH in the same review. After analyzing differences across each modality, these reviewers conclude that dosimetry depends on the specific particle type. In the review conducted by Siddique et al.,^13^ for instance, they state, “There is a need to investigate the dosimetric properties of various radiation modalities used in FLASH RT, including electron beams, proton beams, and x‐rays. Each modality may have distinct dosimetric characteristics, and understanding their behavior in the context of FLASH RT is crucial for optimizing treatment planning and ensuring accurate delivery”. If dosimetry depends on the particle beam used, the literature reviews about dosimetry in each modality should be split up.

Each modality has a multitude of irradiation devices available for study. The most advanced choice of modality is protons, which have already begun clinical trials. The main issue with protons lies in their availability to researchers. There are very few centers in the US, with the National Associate for Proton Therapy reporting 46 centers at the time of writing.^14^ Only some centers can achieve FLASH dose rates, limiting the abundance. Heavy ions are even rarer. Photons are available, but FLASH photons predominantly consist of kilovoltage sources. This energy level cannot effectively treat humans since no skin‐sparing effect occurs. The last modality available is electrons. Electrons are safer due to lower penetrability than the other modality types. Safety is not as much of a concern even in FLASH dose rates compared to other modalities. Obtaining an electron FLASH source has been made quite simple. Current clinical LINACs have been able to undergo conversion processes to increase the dose rate. Schüler et al.^15^ pioneered this new technology by modifying a clinical LINAC to deliver FLASH by tuning the LINAC and utilizing the inverse square law by placing the subjects into the gantry head for irradiation. Placing subjects this way was only a preclinical solution since the gantry head is too small for humans. Other authors, such as Rahman et al.^16^ and Sloop et al.^17^ improved upon these results. In these methods, FLASH dose rates occurred at the isocenter by altering more components of the LINAC in addition to tuning.

The significant barrier to entering electron FLASH research is having a decommissioned LINAC. No commissioned clinical LINAC can be converted for FLASH due to patient safety concerns and vendor contract violations.^17^ Some vendors, such as Varian, have their own LINAC conversion package. Sloop et al.^17^ explain that the manufacturer safeguards modifications. Using an unapproved conversion violates their service contract. This results in the termination of any contract for that LINAC system.^17^ Independent conversions will be able to perform preclinical investigations only.

Opposed to modifying a LINAC for FLASH, many FLASH electron‐specific devices are on the market. Most are intraoperative radiation therapy (IORT) devices. Examples are the Oriatron eRT6, the Mobetron, and the electronFLASH research system. These LINACs have designs that are different from those of standard medical LINACs.^18^, ^19^ The electronFLASH system located in Italy has a shorter waveguide, different energies (5 and 7 MeV), toroid sensors in the waveguide, and several other differences.^19^ This research device can achieve larger field sizes than modified LINACs and more significant dose rates up to 10,000 Gy/s.^19^ The Mobetron is made for IORT, where the applicators are placed directly in the patient, and large single‐dose deliveries are conducted.^18^ Additionally, this device is self‐shielded and limited to 12 MeV beams.^18^ These devices are well established. However, modifying LINACs to achieve electron FLASH dose rates has many advantages. These new devices require new installations, staff training, enormous upfront costs, and sometimes new shielding. Except for self‐shielded models, the electron‐specific devices and modified LINACs will take up a vault. Currently, the modified systems are purely preclinical. If these platforms mature, they will be the easiest to implement in the clinic with necessary safety features and processes established. LINACs could have separate conventional (CONV) and FLASH modes built into the same system. This is not an unusual thought. Current clinical LINACs already have several modes consisting of photons, electrons, flattening filter‐free, and stereotactic capabilities. The current design switches between modes via the movement of mechanical components, changing currents, and altering waveforms. The modifications to the LINACs to enable FLASH are no different. With this method, vault space could effectively be saved. Many generalized LINAC procedures can still apply to these systems, and workflows can remain similar. There is one significant issue with converted LINACs. Transmission ion chambers are no longer compatible with FLASH due to the recombination factor shifting out of clinical tolerance. For a CC13 ion chamber, Poirier et al.^20^ measured the TG‐51 recombination correction to be 1.06. This value is outside of clinical tolerance.^20^ Similar issues along with saturation have been observed with the transmission chambers.^15^, ^17^, ^21^ However, many studies have emerged to create new control methods and attempt to fix the current. Part of this review summarizes the new beam control mechanisms tested.

The other half of this review focuses on electron FLASH dosimetry, as there is a need for modality‐specific FLASH dosimetry reviews. Aside from modality specification, a different approach is used for the overall organization of the dosimetry portion. In previous reviews, dosimeters were referred to more broadly by class. Additionally, much space was used to discuss the operation principles of these classes. In this review, dosimeters are organized in terms of measurement goals, consisting of the measurement of total absorbed dose (TAD), beam characteristic, dose per pulse (dose per pulse, or reading per pulse [RPP] if uncalibrated), and counting pulses. Only detectors capable of the direct measurement of each goal are included. Specific information is also provided, such as calibration and tested irradiation conditions. The dosimeters confirmed to achieve these measurement goals are stated in tables by referring to the specific models tested. Detectors of the same class can have different operational abilities and architecture. Referring to models specifically helps illustrate what models have been examined and in what conditions they have been validated for use. However, since the operational principles and properties of general classes of detectors in FLASH have been sufficiently covered previously, this information is omitted from this report.

The organization is further optimized by following the recommendations of Erol et al.^22^ for structuring a review article. The literature survey conducted here is systematic. The steps in finding the papers reviewed is clearly outlined for the reader in the methods section. The results section has exclusion information of papers in the search and has the actual review itself. This review structure gives the reader a clear path to find these papers.

This review article is for institutions setting up or possessing a FLASH system by modifying a clinical LINAC to deliver electrons. Readers will learn about dosimetry availability for each measurement goal. The detectors achieving measurement goals are discussed by model type, and the conditions they have been tested are clearly outlined. This review provides calibration procedures for some detectors and current work on beam control systems. The results of this review are outlined in tables. One table outlines the pros and cons of every detector examined. These pros and cons are only those discussed in the reviewed reports. This table provides complete transparency regarding the appropriate use of detectors.

## 2 METHODS

**FIGURE 1:**
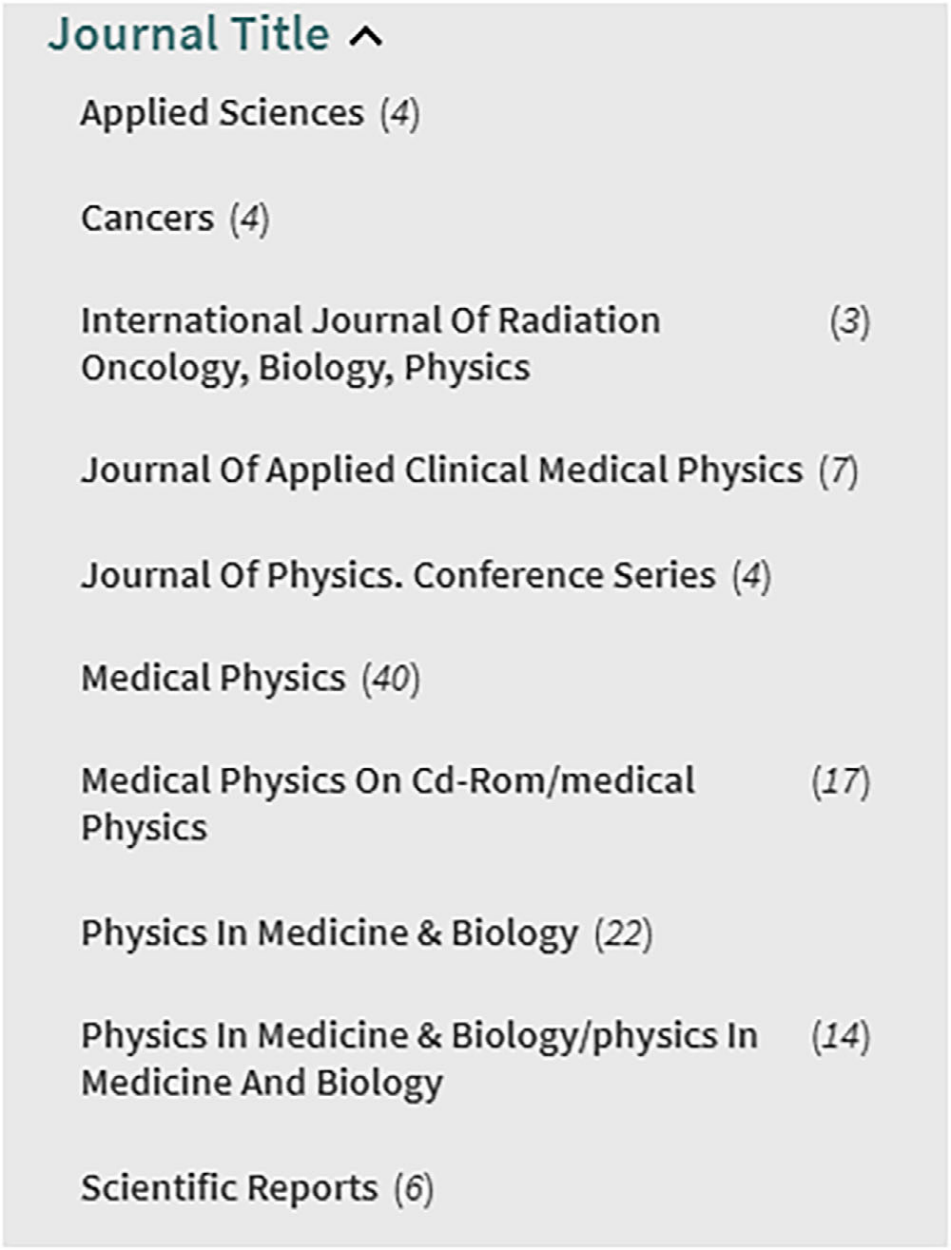
Journals from VCU libraries that were examined in this review. The number on the side corresponds to the number of results found in each journal. These were all results of the literature search detailed later in this section.

The recommendations from Erol et al.^22^ were followed for this review. The databases utilized were Google Scholar and our university's library website, Virginia Commonwealth University (VCU). Affiliates of VCU are granted free access to various journals through the VCU Libraries website. Figure 1 depicts the journals found with relevant articles from this systematic search. The library's entire database of journals is too large to be detailed in this report. A complete list can be found here: https://guides.library.vcu.edu/az.php (as of November 2024).

A preliminary search helped formulate a keyword scheme to use for this review. Upon much refinement, the chosen keyword scheme was *Electron & (FLASH OR “FLASH‐RT” OR “FLASH irradiation”) & (UHDR OR “ultra high dose rate” OR “ultra‐high dose rate”) & Linac & Dosimetry & Detector*. This keyword scheme got copied and pasted into each website's search bar. Many articles refer to FLASH and ultra‐high dose rate in several different ways. Several unique options were included for these terms. We consider this combination provided enough number of articles with minimal risk of overlooking significant publications.

From the same preliminary research on this topic, many articles credit Favaudon et al.^2^ as the pioneer in modern FLASH radiotherapy research. Due to the extensive credit to this study and the convenience of the publication date being precisely 10 years ago, the date range was set to be within the last 10 years. Aside from setting a date range, both websites had a few specific settings. VCU libraries allow users to pick material types in which “articles” were selected. They also allow users to select only open‐access and peer‐reviewed options, which were not selected for this review. On Google Scholar, the boxes corresponding to including results with citations and patents were deselected.

The next step was to sift through the results. This systematic review aimed to return articles that modified a LINAC to deliver electrons at FLASH dose rates discussed dosimetry characterization, and/or provided insight into how they established beam control. A two‐step process was performed to find articles returned from this search that fit the criteria. In the initial refinement stage, the title and abstract of each full article found were read. An issue with this was that articles typically do not include the irradiation device model in the title/abstract, and the device is embedded in one of the body sections instead. Journals only sometimes allow product names in the title and abstract. If it needed to be clarified from the title and abstract that a modified LINAC was being utilized, the article would be skimmed to find the irradiation device. Unfortunately, skimming articles in full added significant time to the search. Repeated articles from the two websites were removed in the second refinement stage. Once all articles were unique, each was read fully to determine if they belonged in this review. The results section further details specific reasons for exclusion in each stage.

## 3 RESULTS

### 3.1 Article selections

**FIGURE 2:**
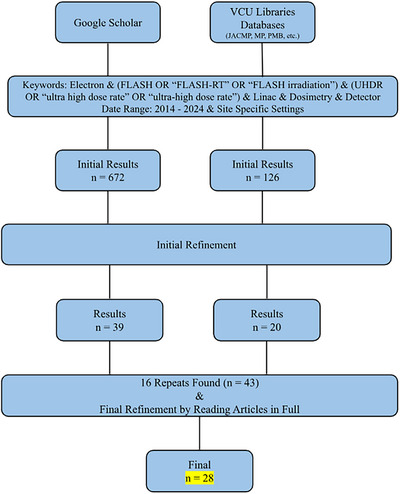
Flowchart depicting a summary of the search conducted. The chart reads top to bottom, with each website in a column. This search resulted in 28 papers for this review.

Figure 2 depicts a flowchart summarizing the number of papers found versus those included in the review. The systematic search found 28 relevant papers for this review.

There were several major reasons why articles were not included in this report. In the initial stage, many studies did not involve a modified LINAC but rather an electron FLASH‐specific device. Another main reason was that articles did not involve electrons but other modalities like protons, heavy ions, or photons. Some articles involved potentially valuable devices for FLASH but were only testing them in CONV mode or simulations. Other exclusions were due to undesired formats like thesis works, presentations, other reviews, non‐reviewed preprints, or partial texts. Sometimes, the same article would appear on the same site multiple times because it was published in multiple journals. Lastly, a few articles had access restrictions.

After removing repeats from both site's results, the articles were read in full and omitted if they did not fit the criteria. Papers were excluded if they did not go into enough detail about dosimetry or control to be included in the tables. A lack of detail could occur because the article was primarily biological. In these studies, the irradiation device and dosimetry were irrelevant to the objective. Alternatively, due to many articles being published by the same groups with the same irradiation platforms. In successive publications to the establishment of the converted LINAC, the original paper was referenced instead of reexplaining the control and dosimetry methods. In this stage, articles were removed if the previous criteria from the first refinement were missed. In a few studies, the dosimetry failed, and detectors were not recommended. These were removed if the article did not additionally speak on control. This report aims to provide a comprehensive list of detectors available for use. Lastly, a few studies involving radiation surveying or failure modes and effects analysis only were excluded.

### 3.2 Dosimetry

A portion of the literature survey resulted in the findings of many detectors examined in FLASH. They have been confirmed to be capable of measuring various quantities with modified LINACs. Table 1 organizes these findings. Dosimeters are grouped into the general class and then are specified by separating each model into rows. The article was excluded from this table if the model was not provided. The calibration, comparisons to other dosimeters, and irradiation conditions were included. Conditions other than those in the table have yet to be examined, and readers should use caution when entering that domain. To make the table more effective, illustrated are the measurement goals each model was examined to collect. Measurement goals consist of TAD, beam characteristics (BC), DPP, RPP, and PC.

**TABLE 1:** Electron FLASH dosimetry with modified LINACs.

Detector class	Detector model(s)	Reference	Uses^a^	Calibration	Compared against?	Agreement? (Y/N)	Modified LINAC	Energy (MeV)	DR/repetition rate	DPP	Source‐detector distance	FS and applicator	Comments
**Diamond**	**microDiamond (PTW, SN122323, and SN122325) read with Unidos webline electrometer (PTW)** ^b^	Dal Bello et al.^23^	TAD, BC (profiles, output vs. gantry angle, and PDDs)	Cross‐calibrated against EBT3 films	EBT3 Gafchromic Film (calibrated 0.5‐20 Gy in 16 MeV CONV), HD‐V2 films (Ashland, calibrated 1–100 Gy in CONV), IC profiler (calibrated in 16 MeV CONV), and corrected Advanced Markus IC. Mainly comparing between CONV and FLASH characteristics however	Y	Varian Truebeam SN 1001	16	Average DR at isocenter = 256 Gy/s, tested upstream at SSD = 59 cm for DR = 837 Gy/s // Tested 20–200 Hz	Max DPP at isocenter = 1.28 Gy/pulse	Isocenter (in 3 cm depth in RW3 phantom)	Mostly 10 × 20 cm^2^, tested 4 × 4–25 × 25 cm^2^	Only SN 122325 was found to be linear up to 1.5 Gy/pulse; the other was not. Films were used to verify PDDs/Profiles/Output, IC Profiler was only for profiles, and Advanced Markus chamber was only for output factors.
**Diode**	**EDGE Diode SNC 1118 read with modified PC electrometer from SNC 1014**	Rahman et al.^24^	TAD, BC (PDDs), DPP	Cross calibrated against film dose measured at 2 cm depth in solid water	TAD—EBT‐XD Gafchromic Film and Ion Chamber in bremsstrahlung tail // BC—EBT‐XD Gafchromic Film // DPP—Exradin W1 scintillator	Y (within a specific ranges)	Varian Clinac 2100 C/D	10	30–180 Gy/s // 360 Hz	0.05–0.78 Gy/pulse	Isocenter	N/A	Modifications to electrometer performed by manufacturer. DPP was able to be measured, but the USB connection was not fast enough to transfer the data in real time
		Sloop et al1^17^	DPP	N/A	N/A	N/A	Varian 2100EX and Trilogy	10	432–72 Gy/s at isocenter // 360‐30 Hz	∼ 1.2 Gy/pulse	Isocenter in 5 mm depth in solid water	Open	Compared two LNAC systems
	**EDD 2–3G Diode (IBA) counted with microcontroller** ^b^	Lempart et al.,^25^ Konradsson et al.^26^ (same system)	PC	N/A	External monitor chamber connected to a digital oscilloscope (RBT2004 Rohde & Schwarz) (Konradsson)	Y	Elekta Precise	10	Tested from 30–300 Gy/s // 200 Hz	Tested from 0.18–1.9 Gy/pulse	Isocenter (off axis), also 19, 37, 53 cm	10 × 10 cm^2^ applicator and 40 × 40 cm^2^ open	At the edge of the field. MCU measures the rising edge of the signal. Different MCUs were used (Arduino Due and Atmega 238)
	**EDP 20–3G Diode (IBA)** ^b^	Lempart et al.^25^	TAD	N/A	EBT3 Gafchromic film	Linear with DPP and dose	Elekta Precise	10	Tested from 30–300 Gy/s // 200 Hz	Tested from 0.18–1.9 Gy/pulse	19, 37, and 53 cm	40 × 40 cm^2^	Results show this detector could be calibrated to measure dose in FLASH
**TLD/OSLD**	**3 Types of TLDs: LiF:Mg,Ti, LiF:Mg,Cu,P, CaF2:Tm // 2 Types of OSLDs: Al203:C, BeO all from Luxel in 3D PLA holder**	Motta et al.^27^	TAD	Read background signal, irradiated with a built‐in known source on the reader for a set time, read again. Repeat for several times to make a calibration curve (one for low dose and one for high dose). Relate to dose in CONV electron beams.	N/A	N/A	Varian Truebeam	16	DR: 200–3 × 10^5^ Gy/s // 200 Hz	8.3 × 10^−4^–1.255 Gy/pulse (isocenter)	Isocenter (in 3 cm depth in RW3 phantom)	10 × 10 cm^2^	N/A
	**Round TLD‐100 LiF:Mg:Ti chips from Thermo Fisher**	Jorge et al.^28^	TAD	Calibrated in terms of dose to Co‐60 at institute of radiation physics. Traceable to METAS (primary standard)	EBT3 Gafchromic Film and Alanine (calibrated in CONV)	Y, closest agreement to film, farther from expected than Alanine	Varian Trilogy	16.6	Average of 150 Gy/s // 90 Hz	Average of 1.5 Gy/pulse	19.2 cm	4 × 4 cm^2^	Applied corrections at time of reading with uncertainty of 2.9%
**Radio—luminescence**	**Exradin W1 Scintillator** ^b^	Ashraf et al.^29^	TAD, DPP	Measured 2 calibration factors: gain and Cherenkov to light ratio (Frontbonne et al. 2002)	TAD—Gafchromic film	Y	Varian C‐Series	10	50–350 Gy/s // 60–360 Hz	0.3 ‐ 1.1 Gy/pulse	Range tested: 77–130 cm	Small circular FS 1 cm–4 cm and calibration in 40 × 40 cm^2^ open field	Radiation hardness issues
	**Exradin W2 Scintillator (1 × 1 mm and 1 × 3 mm) with modified MAX SD signal processor** ^b^	Oh et al.^30^	TAD	MAX SD finds a Cherenkov to light ratio from the green and blue color channels	EBT‐XD Gafchromic film and Advanced Markus Chamber plane parallel	Y, within 2%. Markus agreed with film within 5% up to 0.4 Gy/pulse	Varian Clinac 23EX	16	180 Gy/s at isocenter // 18–180 Hz	Max 3.6 Gy/pulse (for one pulse)	Range tested: 61.6–132 cm	10 × 10 cm^2^ no applicator	Vendor of MAX SD modified the device for them. The 1 × 3 mm was only linear up to 0.4 Gy/pulse (multiple pulses) or 0.8 Gy/pulse (one pulse). 1 × 1 mm was linear with dose to the max of the system. Optical fiber is damaged 2% per kGy
	**DoseOptics iCMOS Camera**	Rahman et al.^31^	TAD, BC (surface profiles and PDDs), RPP	Found conversion factor from counts to dose with EBT‐XD film measurements. Corrected for camera angle with MATLAB transformation	EBT‐XD Gafchromic film	Y—TAD—within 3% after ramp up // BC—Scintillation agreed better by 10% in gamma analysis (3%/3 mm)	Varian Clinac 2100 C/D	10	50–400 Gy/s // 60–360 Hz	0.18 ‐ 0.91 Gy/pulse	Camera out of field (tested two configurations)	40 × 40 cm^2^	Camera was time gated to the KlyV signal from the LINAC. Captured Cherenkov emission from a solid water phantom and scintillation from a rare earth screen on top of a phantom. Scintillation was only confirmed to work properly in all aspects. Could resolve individual pulses.
		Ashraf et al.^32^	BC (3D dose distribution), RPP	N/A	EBT‐XD Gafchromic film	Y—Reconstructed Profiles (besides one) agreed 100% with gamma 2%/2 mm. Reconstructed PDD agreement besides buildup	N/A	N/A	50–70 Gy/s // 60 Hz	N/A	Camera out of field	40 × 40 cm^2^ and 1.5 cm circular field	Used bandpass filter to ignore Cherenkov. Could reconstruct individual pulses (time‐gated). Imaged water tank with quinine sulfate solution. Processing steps required to reconstruct median filter, flatfield, alignment, symmetrization, and Abel inversion
	**CMOS Camera—Quad Channel CXP‐12 GigaSens (Concurrent EDA) (with and without intensifier)**	Rahman et al.^33^	BC (surface profiles), RPP	Dark field image subtraction, flat field correction, 3D median filtered. Did not calibrate to dose.	N/A	N/A	Varian Clinac 2100 C/D	10	Mean DR = 240 and 260 Gy/s for the 1 and 1.5 cm cutout //360 Hz	0.66 Gy/pulse for 1 cm cutout, 0.72 Gy/pulse for 1.5 cm cutout	Camera out of field	1 and 1.5 cm diameter cutouts	The camera was gated to the SYNC signal from the LINAC. SNR increased with intensifier, but SR decreased. Was able to resolve individual LINAC pulses but not in real time (required calibrations after). Cherenkov profile was larger than the actual field.
	**HC‐120 Series PMT (Hamamatsu) coupled to optical fiber (Thorlabs) fed into a BNC cable, digitized with 3000 series Picoscope (PicoTechnology) interfaced with MATLAB** ^b^	Rahman et al.^31^	PC	N/A	N/A	N/A	Varian Clinac 2100 C/D	10	50–300 Gy/s at the isocenter // 60–360 Hz	0.81 Gy/pulse	Edge of field at isocenter distance	N/A	N/A
	**Coincidence scattered radiation detector (DoseOptics) (Remote trigger unit RTU)** ^b^	Ashraf et al.,^29^ Rahman et al.^16^ (same system)	PC	N/A	N/A	N/A	Varian Clinac 2100 C/D	10	50–300 Gy/s at the isocenter // 60–360 Hz	Up to 1.1 Gy/pulse	Many distances	Many FS	Two BGO scintillators and two silicon photomultipliers
	**Hyperscint RP100 Scintillator system** ^b^	Poirier et al.^20^	TAD	Software system prompts user for measurements to remove Cherenkov, background, and relate to dose from CONV irradiation	EBT‐XD Gafchromic Film and CC04 Pinpoint Ion Chamber IBA (relative)	Y within 4% of films	Varian 21EX	16	100 Gy/s at 55 cm SSD, 34 Gy/s at isocenter // 18–180 Hz	Tested 0.55, 0.51, 0.27, 0.20 Gy/pulse	Tested 55, 60, 80, 100 cm SSD	N/A	Dose calibration must be done every time the detector is disconnected from the reader. Software has a PC feature as well, but the readout system causes errors
**Alanine**	**Alanine pellets read with Bruker EPR Spectrometer**	Jorge et al.^28^	TAD	Calibrated in CONV on an Elekta Synergy against ion chamber traceable to METAS	TLD and EBT3 Gafchromic film	Y, alanine was closest to the expected dose.	Varian Trilogy	16.6	Average of 150 Gy/s // 90 Hz	Average of 1.5 Gy/pulse	19.2 cm	4 × 4 cm^2^	Used a custom phantom to test film, TLD, and alanine together. Lacked absolute reference.
**Ion chamber**	**IC Profilier Sun Nuclear** ^b^	No et al.^34^	BC (profiles)	Calibrated with 5 V on the grid to avoid saturation	EBT‐XD Gafchromic Film	Y	Varian Trilogy	17.5	36.82–112.83 Gy/s // 180 Hz	0.21 ‐ 0.63 Gy/pulse	59–100 cm SSD	Tested 3, 10, and 20 cm circular custom‐made cutouts	N/A
		Poirier et al.^21^	BC (profiles)	Calibrated with the lowest voltage possible to avoid damage	N/A	N/A	Varian 21EX	16	2067 Gy/min at the monitor unit chamber	N/A	165 cm SSD to reduce signal	N/A	N/A
	**Wellhofer Model CC01** ^b^	Kyle et al.^35^	TAD	Calibrated in CONV 9MeV beam	Previously verified against film, scintillator, and OSLD (see comment)	Y	Varian Truebeam	10	142 Gy/s and 59 Gy/s (depending on SSD) // 360 Hz	N/A	62.5 and 100 cm SSD	40 × 40 cm^2^ or 20 × 20 applicator	For verification of the IC, they reference an abstract presented at AAPM 2021 by Duzenli et al.
	**Advanced Markus Plane Parallel (PTW 34045)** ^b^	Oh et al.^36^	TAD, BC (Profiles and output factors)	Found charge to dose curve and corrected recombination using models (see comment)	EBT3 Gafchromic Film and OSLDs (AL2O3:C)	Y, and the film/OSLDs were within 3% agreement	Varian Clinac 23EX	16	500 Gy/s at 59 cm	1.27 Gy/pulse at isocenter	Range tested (isocenter, 59 cm, etc.)	10 × 10 cm^2^, no cone	Tested models for correcting for recombination (Boag with and without extension and logistic, which worked the best). Found linearity up to 0.1 Gy/pulse then saturation.
	**CC04 Pinpoint IBA** ^b^	Poirier et al.^21^	Estimate TAD (see comment)	N/A	EBT‐XD Gafchromic Film	Y	Varian 21EX	16	2067 Gy/min at the monitor unit chamber	N/A	SSD = 100 cm, 10 cm depth	25 × 25 cm^2^	Found a ratio of the signal in the bremsstrahlung tail from CONV to FLASH, applied this ratio to the dose in standard conditions in CONV. Measured a recombination factor of 1.06, outside of TG‐51 tolerance.
**Film**	**Gafchromic EBT‐XD Ashland** **Gafchromic EBT3 Ashland**	Oh et al.,^30^ Cetnar et al.,^37^ No et al.,^34^ Petusseau et al.,^38^ Rahman et al.,^31^ Sloop et al.,^17^ Jorge et al.,^28^ Konradsson et al.,^26^ Garty et al.,^39^ Adrian et al.,^40^ Deut et al.,^41^ Konradsson et al.,^42^ Kyle et al.,^35^ Lempart et al.,^25^ Levy et al.,^43^ Szpala et al.,^44^ Xie et al.^45^	TAD, BC (PDDs and profiles)	Calibrated in CONV in a relavent dose range, usually following output check with ion chamber	Considered gold standard. Some compared to TLD, OSLD, Alanine, and ion chambers	N/A	Many different LINACs utilized	Range of energies	CONV–600 Gy/s observed	CONV–27 Gy/pulse observed	Range of setups		N/A
	**OC‐1 OrthoChromic film for > 20** **Gy**	Garty et al.^39^					Varian Clinac 2100C	9	> 600 Gy/s // 180–360 Hz	>1–3 Gy/pulse	20–30 cm SSD	2 cm diameter beam projected at 20 cm SSD	

^a^ Uses of detectors are one of the following options: Total absorbed dose (TAD), beam characteristics (BC), dose per pulse (DPP), reading per pulse (RPP, same as DPP but not calibrated), or pulse counting (PC).

^b^Signifies detector systems that could measure online (i.e., in real‐time). Note that the detector may be in real‐time, but it does not have this symbol due to the readout system restricting its temporal resolution.

BC includes percent depth dose (PDD) plots, cross‐sectional profiles (flatness/symmetry), output versus gantry angle, and so forth. BC identifies the converted beam's spatial output, typically in a phantom. These characteristics are essential for dose calculations with treatment planning software and are necessary if the FLASH beam is ever used on subjects. Due to the vast diversity of BC, Table 1 specifies these in their corresponding rows.

Due to the limited work on measuring these quantities, DPP, RPP, and PC are challenging dosimetric topics with FLASH beams. These quantities are not measured in CONV mode, though they are known. In an AAPM summer school presentation, it was stated that a typical Varian LINAC (very general statement) has a DPP of 0.03–0.06 MU/pulse.^1^ This is a very small amount, and thus characterizing this quantity is not essential in CONV mode. In FLASH, the DPP is very large, raising the importance of measuring it. The RPP and PC are closely related quantities to DPP. RPP is DPP without calibration to dose. PC only measures how many pulses are delivered but does not provide pulse height information (i.e., dose). In Section 3.2, it is shown that the popular method to control FLASH LINACs is by counting pulses instead of monitoring by dose. In this situation, it is essential to be able to characterize the DPP for an approximate prescription. As shown in Table 1, there are many detectors capable of measuring DPP (or RPP). Note that not all of these detectors can resolve DPP online. Some detectors require post‐processing procedures first, where the DPP information is extracted later. In this field, the need for online detectors is becoming crucial. To clearly illustrate which detectors are online, detectors are marked with a b sign after in Table 1.

The pros and cons of all of the detectors in Table 1 are presented in Table 2. This table aids in choosing a detector by providing further considerations. The pros and cons in Table 2 are only those discussed in the articles found in this review. Some of the general pros and cons of detector categories can be found in other review articles for FLASH dosimetry, such as Ashraf et al.,^11^ Romano et al.,^12^ and Siddique et al.^13^ Specific information regarding detector properties, how they function, and their characteristics is beyond the scope of this review.

**TABLE 2:** Dosimeter pros and cons.

Detector	Uses^a^	Pros	Cons	Resource(s)
**Gafchromic EBT‐XD Film**	**TAD BC**	Widely utilized DR independent Better dynamic range and lower uncertainty than EBT3 Calibration in CONV Can be cut and shaped to desired size	Large uncertainty (up to 4%) Passive and requires read out time. Usually, 24 hrs. to a week Many calibration measurements required with known dose delivered Orientation effects	Oh et al.^30^ Cetnar et al.^37^ No et al.^34^ Petusseau et al.^38^ Rahman et al.^31^ Sloop et al.^17^ Jorge et al.^28^ Konradsson et al.^26^ Garty et al.^39^ Adrian et al.^40^ Deut et al.^41^ Konradsson et al.^42^ Kyle et al.^35^ Lempart et al.^25^ Levy et al.^43^ Szpala et al.^44^ Xie et al.^45^
**Gafchromic EBT3 Film**	**TAD BC**	Widely utilized DR independent Calibration in CONV Can be cut and shaped to desired size	Large uncertainty (up to 4%) Passive and requires read out time. Usually, 24 hrs. to a week Many calibration measurements required with known dose delivered Worse dynamic range and higher uncertainty than EBT‐XD Orientation effects	
**OC‐1 OrthoChromic Film**	**TAD BC**	Higher saturation level to dose than EBT3 Better high dose rate performance than EBT3 and EBT‐XD Utilize for high dose measurements	Requires reflection mode scanning	Garty et al^39^
**microDiamond PTW**	**TAD BC**	Linear response up to 1.5 Gy/pulse Could be used as primary detector for beam characterization (SN 122325) PDDs and profiles agreement with EBT3 Gafchromic film Could be used to measure output factors and output vs gantry angle	Above 1.5 Gy/pulse is uncertain Required cross calibration There is another more optimized model from PTW flashDiamond	Dal Bello et al^23^
**Edge Diode SNC 1118 (N‐Type)**	**TAD** **BC** **DPP**	Linear response DR, energy, and DPP independent Good temporal resolution (0.1 ms) Comparable to IC in terms of precision No recombination correction Agreement with EBT‐XD Gafchromic film to within 3% Energy independent within 4% of film Good SR Dose conversion given Agreement with W1 scintillator within ‐7 to 5% Tested during ramp up as well (various DPP) and had 4.5% error bars (only due to threshold setting with device) Independent of repetition rate	Film has a higher SR Lost 0.4% per kGy of sensitivity Needs further characterization Electrometer requires customization (simple and provided by manufacturer though) Not tested above 0.78 Gy/pulse and 180 Gy/s USB not fast enough to transfer data in real time	Rahman et al^24^ Sloop et al^17^
**EDD 2–3G Diode IBA**	**PC**	Agreement with an external monitor chamber (monitor chamber was able to measure DPP with large uncertainty after corrections applied) Was able to control delivery by # pulses and was accurate every time (> 1000 trials)	Required construction of a MCU to actually count No information on DPP	Lempart et al.^25^ Konradsson et al^26^
**EDP 20–3G Diode IBA**	**TAD**	Can be used to tune a LINAC to FLASH Could be calibrated to measure output in FLASH Linear with dose Linear with DPP compared with film	Study did not mention the calibration procedure	Lempart et al.^25^
**Ion Chambers**	**TAD BC**	Can provide accurate data for profiles in UHDR Can be used to approximate DR Logistic model can accurately track saturation	Saturation and recombination effects at UHDR outside of clinical tolerance Requires many verification tests alongside other detectors first Requires models to correct for recombination Saturation at “low” DPP Profilers need to be used at a low voltage and far away from the source to compensate	Kyle et al.^35^ Oh et al.^36^ Poirier et al.^21^ No et al^34^
**W1 Scintillator Exradin**	**TAD** **DPP**	Agreement with film measurements Independent of DR up to 360 Gy/s, DPP up to 1.1 Gy/pulse, and FS Microsecond temporal resolution	Major damage issues of 16.2% loss / kGy (declines after accumulation of more dose) Recalibration required almost every use Must account for Cherenkov production Tedious calibration (10 measurements, 2 factors)	Ashraf et al^29^
**W2 Scintillator Exradin with MAX SD**	**TAD**	Agreement with 2% of film MAX SD corrects for Cherenkov itself Smaller model linear with dose up to the max settings of the LINAC No pulse repetition frequency non‐linearity	MAX SD required modifications by vendor (otherwise saturates) Large volume model saturates at “low” DPP Optical fiber damages 2% per kGy	Oh et al.^30^
**TLDs**	**TAD**	Close agreement with EBT3 film Calibration can be traced to primary standard Reproducible response with different total dose at high DPP DR independent (up to 3 × 10^5 Gy/s)	Requires correction factors for electrons Farther off from dose than film and alanine in UHDR For short SSD, may require energy correction Some sensitization effects	Motta et al^27^ Jorge et al^28^
**OSLDs**	**TAD**	Reproducible response with different total dose at high DPP DR independent (up to 3 × 10^5 Gy/s) Al2O3:C was most precise—agreement with film within 3%	There was a BeO OSLD with a higher sensitivity that was affecting the DR plots (package to package variability) Conflicting results with Al2O3:C across studies Some sensitization effects	Motta et al^27^
**Alanine Pellets **	**TAD**	Close to the expected dose (more than TLD and film in the study) in UHDR Agreement to film within 2% Reproducible	Farther off in dose from film and TLD Study had no absolute reference, results may need further testing Passive, requires EPR	Jorge et al^28^
**Quad Channel CXP‐12 GigaSens (Concurrent EDA) CMOS Camera**	**BC** **RPP**	Can gate to LINAC signal and match repetition rate with capture Excellent SR Temporal resolution to capture each pulse Used in vivo Can maybe obtain estimates of dose, DR, and DPP Picks up respiratory motion effects on DPP when utilized in vivo Worked up to 360 Hz (FPS was matched to 360) Can provide spatial dose distribution info on a pulse‐by‐pulse basis, not just total dose	Low SNR without intensifier Intensifier reduces SR Optical scattering causes measured profile to extend out of the treatment field Requires dark and flat field corrections Requires median filtering Did not convert pixel density to dose No information on DPP	Rahman et al^33^
**DoseOptics iCMOS Camera**	**TAD** **BC**	Linear response Dose rate independent (tested up to 400 Gy/s) DPP independent (tested up to 0.91 Gy/pulse) Scintillation imaging was better than Cherenkov, especially with bandpass filtering Good SR Can reconstruct or directly measure profiles/PDDs per pulse Can achieve high passing rates for certain profiles against film High spatial resolution (1 mm^3)	Cherenkov did not work for PDDs/ profiling due to lateral scatter Technique needs further improvement based on optical alignment, better viewing, speed Must filter Cherenkov signal from Scintillation signal due to energy dependance cintillation requires a quinine sulfate solution Disagreement with films on PDD buildup and some profiles Applicator leakage imaging to remove Lots of processing steps	Ashraf et al.^32^ Rahman et al^31^
**HC‐120 PMT with OF read with 3000 series Picoscope**	**PC**	DR independent Linear with dose (up to 200 Gy tested) and DR (up to 300 Gy/s tested) (360 Hz) Could be used in place of target signal that tunes the beam	No information on the absolute dose in a pulse Was placed at the edge of the field to just monitor the output pulse by pulse Read out system limits temporal resolution (PMT alone has nan‐second resolution)	Rahman et al^31^
**Hyperscint RP100 scintillator system**	**TAD**	Comes with integrated software system that tells exactly how to calibrate and upload measurements for calibration, and auto‐removes Cherenkov/background Software has a built‐in pulse counter Linear with dose up to several thousand Gy < 0.4% signal loss per kGy Agreement to films within 4%	Not all photodetectors are read out simultaneously causes under recording especially if using for dose per pulse (counting) Dose calibration must be performed everyday Temporal resolution is close to that of a 180 Hz beam (2.5 ms)	Poirier et al.^20^
**Remote Trigger Unit DoseOptics**	**PC**	Pulses measured matches expected for low frequencies	Positional dependance with respect to the field PC has some errors in higher repetition rates (maybe due to lag in gating—see control system section) Decay time causes issues	Ashraf et al.^29^ Rahman et al.^16^

^a^ Uses of detectors are one of the following options: Total absorbed dose (TAD), beam characteristics (BC), dose per pulse (DPP), reading per pulse (RPP, same as DPP but not calibrated), or pulse counting (PC).

Gafchromic film (EBT3 and EBT‐XD) were overwhelmingly preferred dosimeters for measuring TAD, BC, and to compare to other detectors. They are well‐understood to be compatible with FLASH. Therefore, specifying conditions in each study would not be useful in the film rows. Film studies are combined into one row per film model to reduce redundant information. In terms of FLASH studies, EBT‐XD is considered superior to EBT3 according to Rahman et al.,^16^ who argue for this due to the improved dynamic range and elimination of certain artifacts. The significant limitations of film are its passive nature and lengthy calibration/readout procedures. Along with film, most detectors found in this survey were passive. This abundance of passive detectors is likely a consequence of the limitations of current online dosimetry using ion chambers.

Many resulting articles still utilize ion chambers under FLASH beams. Instead of characterizing new detectors, some studies hope to correct existing systems. Many recombination models were examined with different chambers. In Szpala et al.,^44^ four models were examined. Like the Poirier et al.^20^ group, they found the standard two‐voltage method inadequate. A Boag model worked well for the lower dose rate range.^44^ The most optimal correction models account for free electrons.^44^ These results are to be taken lightly, as the models were designed for a parallel plate chamber but were applied to a CC13 Farmer chamber. Oh et al.^30^ found that the logistic model worked the best. However, saturation was observed for their ion chamber above 0.1 Gy/pulse. Saturation is a complex issue to fix, which may limit further investigation of correction models. One approach to improving the collection efficiency was presented in Adrian et al.^40^ This article was in the film section of the table since the chamber was used to verify film measurements, but their approach was unique. A farmer ion chamber was placed on the room's ceiling to take advantage of the inverse square law for reducing the dose.^40^ The reading was then correlated to the dose from film.^40^ However, there are most likely reproducibility issues from placing the ion chamber on the ceiling. The correlation to film likely has variation.

If familiarity is desired, another option is to use a diode. Diodes are commonly used for arrays, patient‐specific QA, and to collect test point signals from the LINAC. They are well‐understood and have a workflow similar to that of ion chambers. Resulting articles show that diodes are proving to be useful for FLASH beams. They can measure DPP, sometimes even online.

The most abundant result, aside from films, was the use of radioluminescence detectors. This subgroup is proving to be a valuable candidate for online beam monitoring. Several unique detectors were utilized for these measurements, most notably CMOS cameras. The iCMOS from DoseOptics demonstrated success with scintillation (as detailed in Table 1).^31^ However, Cherenkov measurements exhibited dose blurring in the penumbra, making characterization with Cherenkov less accurate.^31^ The Ashraf et al.^32^ study mentions that Cherenkov has energy dependence, warranting caution for use in FLASH. These cameras could still take on the role of in‐vivo PC and estimation of surface profiles. Scintillation is one of the most promising methods to examine. However, every system comes with issues. The W1 scintillator from Exradin experienced serious radiation hardness issues, losing a maximum of 16.2% response per kGy.^29^ Oh et al.^30^ characterized the new model W2 in FLASH. The degradation in this model has decreased to 2% per kGy.^30^ In the new model, this is caused by the optical fiber.^30^ The Hyperscint RP100 is a very intriguing system. It can provide calibration steps with measurements uploaded to the system, automatic removal of Cherenkov, and count pulses with no additional circuitry needed.^20^ Degradation still exists, but much less than the other systems (< 0.4% per kGy). A significant issue is the periodical under‐recording of doses.^20^ Lastly, the remote trigger unit was used to count pulses. Ashraf et al.^29^ mention that the reasoning for the coincidence was to ensure the measurement was from the beam itself and no other sources, such as cosmic rays or spurious particles^16^ arising from scintillator afterglow or neutron products decaying.^29^ It is unclear if these issues could occur with other scintillator systems.

A group in Italy is researching a different potential solution to online monitoring in FLASH. The articles study the use of sensors (silicon or diamond) to use as a monitor. These articles were not included in the table because they are so unique, and as of now, they do not measure DPP/RPP, BC, or count pulses. Instead, they measure the temporal structure of the output pulses from the LINAC.^41^ Electron and hole pairs are created in the sensor, and voltage is applied to collect the charge and measure the current.^46^ They are so distinct that no device can compare the readings. Deut et al.^41^ studied a silicon sensor from Fondazione Bruno Kessler with eight active volumes. The device is linear with DPP up to 10 Gy/pulse.^41^ This sensor was placed under a high voltage, and the output was connected to an oscilloscope for reading/display.^41^ The RPP could be determined by integrating the signal and dividing it by the oscilloscope's input impedance.^41^ The signal had to be corrected for voltage offset first.^41^ Medina et al.,^46^ from the same group, studied a different material, diamond. Diamond has decreased sensitivity, which is suitable for FLASH.^46^ Added to the diamond are silver layers and a high‐voltage distribution board.^46^ Once constructed, the device works similarly in that high voltage is applied, and the current is read out by an oscilloscope.^46^ This sensor had a drawback in the first few pulses; the collected charge was incomplete.^46^ It turns out that defects of the diamond trap electrons initially, but once the traps are filled, the signal stabilizes.^46^ They hypothesize that this issue could be fixed with pre‐irradiation, but a study should occur.^46^ The group hopes to calibrate to measure DPP in the future, and a comparison study could be conducted. The calibration idea is to correlate the integrated charge measurement to the dose from film or an ion chamber.^41^

### 3.3 Beam control mechanisms

Conventional beam control mechanisms utilize the LINAC's internal monitor unit chambers to halt delivery. Due to specific issues with ion chambers discussed previously, new control systems must be developed. Table 3 outlines the articles that discussed their control system in great detail. The table provides the reader with detectors used, methods, accuracy, issues, and author suggestions for improvements. Note that all detectors in this section are online, which may give a more precise illustration of those available. Be aware that detectors alone cannot count pulses, but complex circuitry systems are developed to assist them.

**TABLE 3:** Beam control mechanisms.

Reference (chronological order)	LINAC	Energy (MeV)	Detector(s)	Methods	Accuracy	Issues	Improvement Suggestions
Lempart et al. 2019	Elekta Precise	10	EDD 2–3G Diode (IBA)	Developed an in‐house control system circuit connected to the LINAC. The diode detects pulses of radiation, and the signal is fed into a two‐stage amplifier and conditioning circuit. Essentially, the photocurrent is converted to 5 V, which is input into the interrupt pin of an Atmega238 microcontroller unit (MCU). This MCU counts pulses by measuring the rising flanks of the signal using a Timer and Interrupt Service Route triggered by the rising flanks. Once the number of pulses counted matches the user‐defined expected, the MCU sends a logic signal to an optocoupler circuit. This circuit prevents trigger pulses from reaching the thyratron of the LINAC, which effectively prevents electron injection into the gun and the magnetron from producing RF for acceleration.	No additional pulses were delivered than expected. This control approach has very little latency.	LINAC stability issues require a warm‐up every 10 min due to the cooling of the machine as a result of the short beam on times in FLASH. It can be fixed with additional tuning. Not monitoring by dose and pulse height variation was observed, limiting accuracy.	Monitor by dose instead. Suggest using the transmission ion chamber (see Konradsson et al. 2024 row). Lower or modulate the gun current to achieve a desired dose with a fixed number of pulses
Szpala et al. 2021	Varian iX	18	Transmission Ion Chamber	Entered MUs in the treatment console, just as in CONV mode.	The stability of the dose for a given MU was satisfactory (standard deviation of charge ∼ 0.1‐0.2 nC). MU chamber was linear with dose and dose rate.	The smallest MU allowed by iX LINAC is 1. Output dependence on the repetition rate (PRF) due to the MU chamber not fully resetting before the next pulse, allowing the same delivery time for all PRF > 200 MU/min (and thus more dose). Samples were placed in the LINAC head. To increase DR further, must move samples farther toward the source. Eventually, samples would partially block the MU chamber	Use an external monitor chamber with recombination correction factors to re‐enable dose rate and steering servos to prevent output issues.
Rahman et al. 2021	Varian Clinac 2100C	10	Remote Trigger Unit (DoseOptics) (coincidence scattered radiation detector)	Developed a control circuit with an Arduino Mega 2560. Control was based on counting pulses (interrupt‐based) up to a preset amount from the RTU or a preset time. RTU (two scintillators and PMTs) detects pulses from outside of the field. The circuit would turn on a gating reed relay until the preset pulses or time was reached. The reed relay output was connected to pins 32 and 34 of the gating switchbox (Varian), effectively asserting an MLC hold‐off signal when the output was on.	Consistent within 1–2 Gy. No other information	Variations in dose per pulse make the system inaccurate. Long execution time of ancillary routine in Arduino firmware (display/keyboard). Interrupt‐based control factored this out.	Need a dose‐based monitor.
Ashraf et al. 2022	Varian C‐Series	10	W1 Scintillator (Exradin) Remote Trigger Unit	Utilized field programmable gate array (FPGA) controller (consisting of a real‐time input/output controller, detectors, trigger signals, and display) programmed with LabView. FPGA performed the following: Synced with the ‘Sync’ signal from the LINAC and generated signals Integrated the signal from the W1 to calculate DPP (dual channel gated integrator) Counted pulses and measure the pulse width from RTU Send a gating signal to the RPM switchbox when the desired dose or pulses are reached	Dose accurate within 1.5 Gy (more accurate for lower doses) & sometimes an extra pulse was delivered at high PRF.	Spurious particles from RTU due to decay time and positional dependence. Relay and gating system lag, causing delivery errors. No pulse width modulation to meet specific dose goals. Damage issues with W1	Modulate the last pulse. Use the gun current pulse for gating (can apply to any LINAC). Use a different scintillation detector with better radiation hardness (liquid?)
Garty et al. 2022	Varian Clinac 2100C	9	N/A	Modified the timer interface card. Replaced GDLY CNT signal (controls delay between electron gun/klystron, which in turn causes beam on or off) with their own. If voltage is applied, they are out of phase (beam off). Used a USB‐CTR08 card to generate control pulses. This accepts the KLY1 signal (klystron pulses) from the controller and turns on output after predetermined pulses (of KLY1). The output pulls up a test point of equal voltage to cause the gun/klystron to be out of phase. Otherwise, this is grounded, and the beam is on.	Pulse height fluctuated 1–2% for lower dose rates but up to 15% for higher. This was out of their control by using a pulse counter.	Pulse height drifted due to room temperature effecting the gun and accelerator. Burn‐in of diodes in the circuit that drives the klystron—replaced. Activation in the LINAC head. Gun and klystron are asynchronous in most irradiations and must wait a certain amount of time for stability.	Develop an alternative beam monitor: The current system uses a monitor chamber and film to measure the pulse height.
Dal Bello et al. 2023	Varian Truebeam	16	N/A	Varian performed the modifications and set up the control system. They set up a prototype patch that modifies the beam generation and monitoring firmware to read the pulse trigger signal and shut off the beam after a preset amount. The dose in a pulse was stabilized by changing the automatic frequency control. An upper limit on the number of pulses allowed was set up. This had two modes where the limit could be increased for tuning purposes. The target could also be inserted, and the target current could be used to characterize pulses.	No differences between the desired and delivered number of pulses	N/A	Real‐time dose monitoring is needed. Independent system
Cetnar et al. 2024	Varian Clinac iX	16	Beam Pulse Counter (Varian)	Varian offers their own LINAC conversion package, FLEX. The package comes with the Varian Beam Pulse Counter, where users can input desired # pulses, and the device controls delivery while providing audio/visual information. No specifics on what the counter is.	N/A (output was reproducible)	Machine head activation following long beam on times. Need to wait to enter the room safely.	Real‐time dose monitoring as opposed to PC. Suggest alternating current transformer placed after the scattering foil to measure a dose.
Oh et al. 2024	Varian Clinac 213EX	16	Beam Pulse Counter (Varian)	Also used the Varian FLEX package but provided more specifics. The package was the same as a pulse counter, which allows the user to deliver a preset number of pulses. The package allows 1–99 pulses to be delivered in one irradiation. The user can choose between 18–180 pulses/s. A required time gap between irradiations is set up as well. CONV beams remain available, but the vendors restrict any clinical use after modification	Out of 165 output measurements, seven deviated from the mean by > 5% and 11 by > 3%. Since using a pulse counter, this was out of their control. This improved after additional modifications to < 1% (see issues)	No active dose control Operation in service mode requires extra QA. One energy configuration is lost to set up the FLASH beam. Output fluctuations existed until tuning the PFN and increasing the mandatory time gap. Fluctuations during warm‐up	Real‐time dosimetry monitoring for FLEX OR Ability to adjust the gun current and RF from the klystron in combination with the existing system to achieve more dose modulation
Konradsson et al. 2024 (upgrade to Lempart et al. 2019)	Elekta Precise	10	External Transmission Ion Chamber (Elekta) mounted on the upper part of an applicator (55 cm from source) EDD 2–3G Diode (IBA)	Developed an in‐house control system circuit. Uses an Arduino Due microcontroller unit (MCU) to send a signal to an optocoupler to disable thyratron trigger signals and thus halt delivery. Signals are sent when one of the following criteria is met: The desired dose is reached as measured by the external MU chamber. The chamber is corrected for temperature and pressure now that is is external, for recombination in FLASH with a logistic model from a previous study of theirs, and normalization to a target dose. A preset number of pulses is reached, as measured by the diode at the field edge (Lempart et al. 2019). The diode signal is digitized and counted by the MCU. This can also be done by connecting an oscilloscope to the external MU chamber. Preset time is reached, t = (n‐1)/PRF, where n = number of pulses desired. Additionally, the system was synced to the pulse‐forming network (PFN) for dose modulation. The PFN has a ramp‐up time, and a user‐selected delay in the beam on signal is introduced.	Each component was examined separately for control and successfully interrupted every time. The MU chamber was linear with dose and DPP. The results of the expected delivered dose were within 5%. This was improved with PFN synchronization to 0.8% Number of pulses between the diode and MU chamber with oscilloscope agreement observed	Transmission MU chamber correction factors introduce uncertainty Cannot fully rule out the possibility of underdosing due to premature beam interruptions Reporting of DPP with modulation using PFN is difficult Results were initial tests only, not long‐term	Need a different beam monitor that is dose rate independent. They are currently investigating a beam current transform (BCT) paced at the exit window. Want to modulate the last pulse instead of the first. Requires software developments and new transmitter components
Deut et al. 2024	Elekta SL 18 MV	10	Unbiased Silicon Diode Sensor	A PC circuit was developed in‐house. The silicon diode measures radiation pulses. The diode signal is converted to voltage with a trans‐impedance amplifier. The resulting voltage signal is then altered by feeding it to a Sallen‐Key filter and amplification. This signal is then passed to a Schmitt Trigger, effectively producing a square 5 V pulse for each radiation pulse measured. These square signals are counted with an Arduino NANO board until a desired amount is reached. It is unclear from here how the LINAC is connected to this circuit to be shut off.	Compare the pulse counter to readings from an oscilloscope relating to the number of pulses delivered. They observed no pulse loss.	N/A	Desire to have a dose‐based monitor as opposed to counting pulses. The group is working on characterizing silicon sensors for online pulse monitoring. This study illustrated the success of these devices. In the future, they will correlate the sensor reading to dose from film measurements for an online monitor.

Abbreviations: BC, beam characteristics; DPP, dose per pulse; PC, pulse counting; PDD, percent depth dose; TAD, total absorbed dose.

This literature survey shows that every institution's control mechanism is different. There are various ways to achieve control, which is possible for any LINAC model. Interestingly, how studies have the LINAC halt the beam is diverse. Ashraf et al.^29^ use the respiratory position management switch box to shut the beam off. There is latency with this approach. The gating switch box method to assert the MLC hold‐off signal used in Rahman et al.^16^ has a similar issue. Some methods exist with minimal to no latency, such as using an optocoupler circuit to prevent trigger pulses from reaching the thyratron.^25^ The Varian FLEX package also has a perfect pulse‐counting control system.^23^ Of course, this is expected from the manufacturer of the modified device. It still begs the question of what exactly they are doing to control. Dal Bello et al.^23^ state that modifications are made to the beam generation and monitoring firmware. Besides this, it is unclear what the pulse counter detector is and how the beam is getting shut off.

Regarding detectors, diodes, and radioluminescence are the popular choices for online monitoring. Regardless of choice, the control accuracy in most situations could be improved. This inaccuracy is an inherent issue with the method for PC studies. In the low pulse range, ramp‐up was observed in LINACs.^16^, ^17^, ^29^ Ramp‐up means that the DPP is initially variable and has a lower intensity. The smaller dose in pulses persists for a few pulses until the DPP increases to a stable average value. This was an issue in the first 4–6 pulses.^16^ Because of the variability in the dose amount in the beginning pulses, simply counting pulses does not always correlate with a known dose in this range. Therefore, PC only can lead to inaccurate deliveries. Sloop et al.^17^ corrected this by tuning the automatic frequency control system. However, PC will still be limited to delivering multiples of the tuned DPP.

Regardless of the ion recombination issues, some articles examine using the MU chamber. Szpala et al.^44^ found that the MU chamber's response depends on the pulse repetition frequency (PRF). The dependency occurred for any PRF > 200 MU/min,^44^ which most studies required to achieve FLASH. Sloop et al.^17^ found a similar result on the PRF dependency of the MU chamber. However, if the PRF is kept constant, a relationship can be found between MU and dose.^17^ Sloop et al.^17^ decided to use caution and only utilize the MU chamber as a second layer of redundancy for their other control system. The control system was created in another study, so this article cannot be found in Table 3. The PRF dependency causes some accuracy issues in the dose. In the study by Konradsson et al.,^26^ they employed an external monitor chamber and applied correction factors, including for recombination. The corrected MU chamber worked well, except for the uncertainty induced by these corrections.^26^ The article recommends a different solution. The only study to attempt dose‐based control without the MU chamber was Ashraf et al.^29^ In this study, the Exradin W1 scintillator measured the dose. However, the control system had latency, which degraded the accuracy. Additionally, the W1 experienced radiation hardness issues, as previously discussed.

A couple of studies opted not to use detectors at all. They examine utilizing internal LINAC signals for control in hopes of improving accuracy. One study used Varian service engineers. In the other study, Garty et al.,^39^ they replace a signal with their own to control the electron gun and klystron phases. By altering the phase, these signals can become asynchronous when it is time to deliver. The phase difference was observed in most of their irradiations.^39^ A delay time was added to achieve stability in each irradiation.^39^ Adding this time in practice is impractical. This idea is the most intuitive, but messing with internal signals must be done cautiously.

No beam control system examined here is the solution. Every author had suggestions for improvement of their system. Similar suggestions were seen across every study. There is a need to monitor by dose and not by counting pulses. New detectors are needed. The sensors studied by Deut et al.^41^ and Medina et al.^46^ are showing promising results to become a potential candidate to fill this void. The other common suggestion is to figure out a way to modulate the gun current to meet dose prescription goals. Many would like to modulate the last pulse in the sequence. Konradsson et al.^26^ cleverly implemented a PFN synchronization to induce a user‐selectable time delay. This enables the utilization of the DPP differences in the ramp‐up. This attempt to modulate DPP needed to be more flexible, and they also recommended modulating the last pulse instead.

#### 3.3.1 Other studies

Aside from Table 3, other studies from this literature survey detailed control systems. These studies were not translated into the table for various reasons. Some needed to provide more details about the system's architecture. Others did not speak about the system's accuracy or provide suggestions for improvement. It may be the case that other articles from the groups with more detail exist. Since the corresponding articles were not picked up from the systematic search, they are outside the scope of this review. This section will briefly discuss the study's unique solutions.

With funding from the Canadian Institute of Health, the BC Cancer Research Institute successfully modified a Varian TrueBeam LINAC to deliver FLASH dose rates.^35^ The article was mainly biological, which may be the cause for the limited control discussion. Regardless, they did mention that the LINAC was controlled in two ways. Scintillator measurements were fed into a microcontroller unit (Arduino), which counted detected pulses.^35^ Additionally, the system was interfaced with the LINAC timer interlock set to the minimum value of 1 s to halt delivery.^35^ The connections from the MCU to the LINAC could be made more explicit.

Before this, Levy et al.^43^ utilized a control method from another article by Schuler et al. Interestingly, Schüler et al.^15^ occurs within the date range examined beginning in 2014 and is published in the Red Journal (ASTRO), which is included in Google Scholar. However, this paper was not found in the systematic search. The interested reader can refer to Schüler et al.^15^ for more details. Levy et al.^43^ provide some insight into the control system. The group utilizes a programmable controller board (STEMlab 125‐14, Red Pitaya) connected to a relay circuit that counts pulses detected by the internal monitor unit chamber.^43^ The board was connected to the respiratory gating system to shut off the beam.^43^ This gating mechanism was utilized in studies like Rahman et al.^16^ and Ashraf et al.^29^ In these studies, there were some latency issues, which most likely occurred here. Outside of control, they employed an external monitor chamber calibrated to film to provide a dose reading per delivery.^43^ The detector appears similar to Konradsson et al.,^26^ but the external chamber did not provide dose‐based control.

Xie et al.^45^ improved upon and simplified the control system proposed by Lempart et al.^25^ Internal signals are used instead of employing an EDD 2–3G Diode (IBA) to count pulses.^45^ The output from the analog signal detector of the beam generation system (either the magnetron current or the pulse forming network) was transferred to a microcontroller (Atmel Corporation) to count pulses.^45^ Once the desired amount was counted (preset by a timer), the controller triggered a relay connected to the Function Keypads interrupt port.^45^ The study claims improved accuracy, increased stability, and simplification improvements compared to Lempart et al.^25^, ^45^

From the same department as an included study, Poirier et al.,^21^ Byrne et al.^47^ discussed advancements to their system. To control their LINAC, they additionally examined the use of the monitor unit chamber. Like other studies, they found linearity between MUs and doses.^47^ Using the same scintillator detector as Poirier et al.,^21^ they determined that 1 MU corresponds to 3–4 pulses.^47^ This way, MUs could approximate the number of pulses delivered since the chamber was saturated.^47^ This study clearly illustrates why PC is insufficient for control. Since the number of pulses varies from 3–4 to achieve the same number of MUs, a certain number of pulses will not correspond to a given dose. The treatment couch was shifted to take advantage of the inverse square law to achieve dose rate modulation.^47^

## 4 DISCUSSION

This review clearly described how it was conducted so that readers could repeat and update the review. We encourage the systematic format of this review. It is essential for readers to understand how the articles were found. When this approach is used, it is recommended to do the search in a couple of steps, with each step enhanced by adding more keywords found relevant in the previous step.

Three tables were created based on an analysis of the articles found in this search. The tables illustrate specific detectors available, the advantages and disadvantages of each model, and how institutions control modified LINACs. It is necessary to emphasize the importance of FLASH studies in reporting every detail of the irradiation conditions. Due to this new realm of dosimetry, clarity is needed regarding the conditions in which detectors have been validated. Parameters outside of specific ranges could change the response of some dosimeters. Certain parameters could broadly impact the biological response. One parameter that may be important to the biological response of FLASH is the instantaneous dose rate.^47^ This parameter was in the original design for the dosimetry table, but not enough studies reported this value.

It turned out many articles from the search used separate electron FLASH devices. Two of these studies examined a potentially revolutionary detector, the flashDiamond (PTW). The flashDiamond is the microDiamond optimized for FLASH beams.^48^ In the Verona Rinati et al.^48^ study, they irradiated the flashDiamond detector with the ElectronFLASH system. They used the detector to measure BCs (PDDs, Profiles) and compare them with EBT‐XD film. They observed agreement with, “somewhat noisier curve was observed with films”.^48^ The flashDiamond could measure a more accurate PDD than film. Kranzer et al.^49^ studied many models of the microDiamond, some of which were flashDiamond prototypes. They saw that “fast, pulse‐resolved measurements with low uncertainty were possible”.^49^ DPP measurements were possible with the microDiamond. It can be inferred that this measurement type is also possible with the flashDiamond. The flashDiamond was an overlooked detector due to the exclusion of these FLASH‐specific devices. A separate review including these platforms could be beneficial. The review should only include electrons, as broader reviews determined the modality dependence on dosimetry.

In terms of the articles found, many dosimetry tools are being tested for electron FLASH with unique modified LINACs. The main takeaway is that dosimetry has, for the most part, regressed. Of all the detectors, the most frequently utilized in electron FLASH is Gafchromic film. Film is a valuable resource for TAD or characteristics measurements in FLASH. The main limitations of film are its passive nature and its uncertainty from scanning and calibration. Real‐time measurements of dosimetric quantities should be a priority of more studies in this category. Many articles provide options for real‐time detector tools, but no detector is without disadvantages.

These systems introduced a new dosimetry category, measuring DPP. This category needs advancement. Many online detectors are available and compatible with FLASH, but not all are being examined for this measurement goal. Some online detectors are capable but need to be calibrated to dose. It is unclear if the calibration procedures were not understood or not being done. The readout systems limited several detector candidates for measuring DPP in temporal resolution. When Rahman et al.^24^ were measuring with the Edge diode, they saw that DPP measurements were possible, and the detector could do this online. However, the USB was not fast enough to transfer the data in real‐time.^24^ Instead, they had to utilize a snapshot mode to extract DPP information from post‐processing.^24^ The information could only be extracted after the entire irradiation.^24^ If the readout system transfer speed were increased, this detector system would be capable of real‐time measurement of DPP. In another study by Rahman et al.,^31^ they showed that the HC‐120 PMT detector system could have nanosecond temporal resolution. However, the readout system using a Pico scope degraded this resolution.^31^ The Hyperscint RP100 system with the integrated software and pulse counter is intriguing. However, Poirier et al.^20^ showed that when measuring individual pulses, the platform sometimes under‐records. The under‐response was due to the photodetectors not being read out simultaneously.^20^ The tool would significantly improve if the system were adjusted to fix this issue. However, the temporal resolution may not have been fast enough regardless.^20^ The readout systems of potentially revolutionary detectors are limiting their temporal resolution. Future research needs to switch gears to improving readout systems' speed and mechanics. If readout systems can improve, the detector tools already found can be utilized. No studies were examining or discussing these issues.

Beam control mechanisms need to be improved the most in this review, where most methods had the limitation of being based on counting pulses. Most studies observed a variation in the DPP, causing uncertainty in the dose with PC techniques. The most significant reason for variability was the ramp‐up in the first several pulses. Sloop et al.^17^ were able to tune the AFC to stabilize the DPP and eliminate ramp‐up. Stability in DPP is a prerequisite if PC will be utilized for control. However, this is still limited because the total dose delivered can only be multiples of the resulting stabilized DPP. Many studies that wish to take this route are working on modulating the dose in the last pulse to reach prescription goals. They can use a fixed DPP with an altered final pulse. This will help to remove the limitation of the prescription being a multiple of the fixed DPP. Only two studies attempted to control based on dose. Ashraf et al.^29^ attempted to use the W1 scintillator detector with an FPGA. However, their system had many limitations regarding lag in gating and radiation hardness of the detector.^29^ They suggest examining different scintillator materials or detectors to use with their system. Luckily, Oh et al.^30^ characterized the newer W2 model in FLASH, finding that the hardness has improved much for this environment. Whether this is the solution to Ashraf et al.^29^ problem is uncertain. Konradsson et al.^26^ used an external transmission ion chamber with many correction factors to control based on dose. The corrections caused too much uncertainty.^26^ The main issue is the new requirement of pulse modulation to achieve a specific prescription goal. This area of research needs to be emphasized, as beam control is necessary if clinical translation continues.

Connections had to be established between the external control circuits and the LINAC. The method with the least latency utilized an optocoupler circuit to prevent pulses from reaching the thyratron.^25^, ^26^ Other methods using a gating switch box or respiratory management have some latency.^16^, ^29^ This latency was caused by the control circuit and the methods by which the beam is halted. In general, lag causes many unacceptable accuracy issues. Some studies report accuracy within 1–2 Gy.^16^, ^29^ Additionally, with some pulse counters, an additional pulse could sometimes be omitted.^29^ In the Petusseau et al.^38^ study, not in this review, they reported that when using three pulses, 8.4 Gy was delivered, but when an extra pulse was let through, they measured 11.2 Gy. One pulse difference is significant due to the abundance of dose in a single pulse in FLASH. These inaccuracies cause dose differences near or greater than the standard fraction size.

Due to the severe inaccuracies experienced in some studies, clinical trials with modified LINACs are not ready. Potential under or overdosing could occur near the values of an entire standard treatment. For accurate methods, authors admit that there is a possibility of underdosing. There is always a chance of faults in the beam interruption methods.^26^ Furthermore, if accuracy is achieved with no error, the new issue of modulating the last pulse to achieve a dose goal still exists. These platforms need to improve accuracy and achieve more flexibility in deliveries. As of now, these systems are merely a preclinical solution. Once an optimal conversion occurs, the challenge shifts to collaboration with the LINAC vendors. Currently, conversions result in contract violations.^17^ There is some optimism that the vendors will alter this. Varian already offers their own LINAC conversion eFLEX package they approve of.^17^ In the future, vendors may allow this as long as it is with their approved method and the conversion is conducted by their service engineers. However, vendor conversion will increase the cost of implementing this platform. The low cost was a significant benefit of these systems.

All authors working on conversion methods have many suggestions for future directions. As discussed, most articles mention the need to develop a method to modulate the last pulse to achieve a dose goal. Regarding the system itself, Ashraf et al.^29^ bring up the importance of standardization in this field. Every control mechanism utilizes complex in‐house circuitry that is only sometimes clearly detailed. These make the recreation of methods complex. Standardization of beam control circuitry could aid in advancing beam control. They suggest using the gun current pulse to gate the beam, which can apply to any LINAC model.^29^ Testing on similar platforms can help advance this research more quickly. Future articles should consider the big picture of how all institutions can utilize their methods. In CONV radiotherapy, all platforms use the same control mechanism of transmission ion chambers. Prescription and dosimetry can be easily translated throughout all clinics.

The main improvements required for these systems involve the detectors. A method yet to be tested on converted LINACs but suggested by many is using a beam current transformer (BCT).^26^, ^37^ Cetnar et al.^37^ suggest placing them after the scattering foil, while Konradsson et al.^26^ want to examine placing them at the exit window. In their discussion, Konradsson et al.^26^ explain that they are widely available for IORT devices like Oriatron eRT6 and the Mobetron. They explain that BCTs can measure induced current from the radiation beam and display it in real‐time. Ashraf et al.^29^ point out that they are limited to smaller field sizes, as the beam needs to travel inside the BCT, which is a small volume. They mentioned that if easy access was available to the waveguide, they could also be placed there. Since these platforms are mainly preclinical, the small field size requirement should not limit any eagerness to test. Instead, Ashraf et al.^29^ suggest using other scintillator materials to utilize their existing control system, such as liquid scintillators. Many scintillator materials are available that still need to be characterized in FLASH. Another model, the W2, shows improved radiation hardness effects.^30^ The Deut et al.^41^ group is taking a brand‐new route and testing sensors for real‐time monitoring. These show promise, and more research is encouraged. More focus should be placed on the readout systems of existing detectors instead of trying new dosimeters. The readout systems limit detector capabilities. If faster readout is achieved, existing detectors could possibly be utilized. No focus on readout was placed in any observed article. The tools may have already been found, but the accessories to enable their use must be improved. Another area for improvement with existing online systems is the need for calibration to be performed. Calibration needs to be carefully examined for existing systems and procedures need to be developed. If this is done, researchers may already have found FLASH‐compatible online monitors.

## 5 CONCLUSIONS

This review article clearly stated why it was necessary. There is abundant FLASH research dealing with modified clinical LINAC beam control systems and dosimetry. The topic of FLASH dosimetry was scaled down to be modality‐specific with a focus on electrons due to differences in dosimetry. The methods for how the reviewed articles were found were clearly outlined. In total, 28 papers were found to have modified clinical LINACs to deliver electron FLASH beams, dosimetrically measure quantities, and/or detail new beam control systems within the last 10 years. The dosimetry portion of the review was effectively organized by matching up dosimeter models to measurement goals. The goals consisted of TAD, BCs, dose (or reading) per pulse, and PC. Detector calibration considerations were outlined when available. Film was observed to be the dominant detector for TAD and beam characteristic measurements. Many exciting new options are under development in the other categories, as they are unique to FLASH. The pros and cons of each utilized detector were outlined in a table to provide readers with additional considerations. Beam control systems were also outlined in a table. Control systems need much improvement, as most only achieve control based on PC mechanisms. The accuracy of these systems is not quite ready for the next stage of clinical trials. Many exciting improvement suggestions were provided. Most suggestions for improvement involve finding new detectors to replace the transmission monitor unit chamber and modulating the dose in a pulse to meet prescription goals. Detector readout systems are also to be significantly improved.

## AUTHOR CONTRIBUTIONS

**Justin DeFrancisco**: Performed the literature search and prepared the draft manuscript. **Siyong Kim**: Designed the study and reviewed the manuscript.

## CONFLICT OF INTEREST STATEMENT

The authors declare no conflicts of interest.

## ETHICS STATEMENT

No AI assistance was used.

## Data Availability

There is no data to share.
